# A Myo-Inositol-Inducible Expression System for *Corynebacterium glutamicum* and Its Application

**DOI:** 10.3389/fbioe.2021.746322

**Published:** 2021-11-15

**Authors:** Nan Lu, Chenglin Zhang, Wenjie Zhang, Haoran Xu, Yuhong Li, Minhua Wei, Jing Meng, Yan Meng, Junzhe Wang, Ning Chen

**Affiliations:** College of Biotechnology, Tianjin University of Science and Technology, Tianjin, China

**Keywords:** *Corynebacterium glutamicum*, expression vector, IolR, iolT1, myo-inositol, 5-aminolevulinic acid

## Abstract

*Corynebacterium glutamicum* is one of the important industrial microorganisms for production of amino acids and other value-added compounds. Most expression vectors used in *C. glutamicum* are based on inducible promoter (P_*tac*_ or P_*trc*_) activated by isopropyl-β-D-thiogalactopyranoside (IPTG). However, these vectors seem unsuitable for large-scale industrial production due to the high cost and toxicity of IPTG. Myo-inositol is an ideal inducer because of its non-toxicity and lower price. In this study, a myo-inositol-inducible expression vector pMI-4, derived from the expression vector pXMJ19, was constructed. Besides the original chloramphenicol resistance gene *cat*, multiple cloning sites, and *rrnB* terminator, the pMI-4 (6,643 bp) contains the *iolR* ^*q*^ cassette and the myo-inositol-inducible promoter P_*iolT1*_. The pMI-4 could stably replicate in the *C. glutamicum* host. Meanwhile, the non-myo-inositol degradation host strain *C. glutamicum*Δ*iolG*Δ*oxiC*Δ*oxiD*Δ*oxiE* for maintaining the pMI-4 was developed. Overexpression of *hemA* ^*M*^ and *hemL* using pMI-4 resulted in a significant accumulation of 5-aminolevulinic acid, indicating its potential application in metabolic engineering and industrial fermentation.

## Introduction

As a non-pathogenic Gram-positive bacterium, *Corynebacterium glutamicum* has been widely used in industrial biotechnology for the production of several million tons of amino acids annually, especially L-glutamate, L-lysine, and L-valine (Hasegawa et al., 2012; Eggeling and Bott, 2015; Wendisch, 2020). Besides amino acids, the current product spectrum that is accessible with *C. glutamicum* comprises organic acids, diamines, vitamins, aromates, and alcohols (Chen et al., 2016; Chung et al., 2017; Becker et al., 2018; Kogure and Inui, 2018; Sato et al., 2020). Benefiting from the genome annotation of *C. glutamicum* and the availability of molecular biology techniques and tools, significant progress has been made in designing production strains by metabolic engineering *via* rational approaches (Ikeda and Nakagawa, 2003; Nesvera and Patek, 2011; Suzuki and Inui, 2013; Wang et al., 2021).

Overexpression of genes encoding the rate-limiting enzymes involved in pathways is one of the most efficient strategies (Nesvera and Patek, 2011; Suzuki and Inui, 2013). Several vectors for gene overexpression in *C. glutamicum* have been developed, such as pXMJ19, pEC-XK-99E, and pDXW-8. Most of these expression vectors are based on inducible promoters (P_*tac*_ or P_*trc*_) regulated by isopropyl-β-D-thiogalactopyranoside (IPTG) (Jakoby et al., 1999; Kirchner and Tauch, 2003; Xu et al., 2010; Li et al., 2020). However, IPTG is toxic to cell growth and expensive, which makes it unsuitable for industrial production (Li et al., 2020).

**FIGURE 1:**
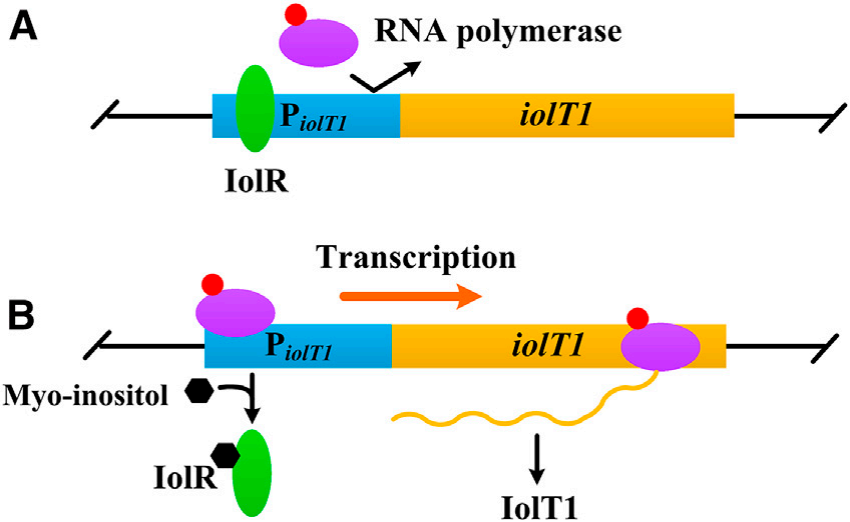
Negative control of *iolT1* transcription. **(A)** No myo-inositol. The repressor IolR binds to the promoter of *iolT1* (P_*iolT1*_) and blocks RNA polymerase from transcribing the *iolT1*. **(B)** With presence of myo-inositol. Myo-inositol as an inducer binds to the repressor IolR, resulting in its dissociation and allowing RNA polymerase to transcribe the *iolT1*.

Myo-inositol, a water-soluble vitamin B group compound, is found to induce transcription of several genes involved in its transport and catabolism (Figure 1), such as *iolT1* (encoding myo-inositol transporter), *iolG* (encoding myo-inositol dehydrogenase), and *iolH* (encoding myo-inositol isomerases/epimerase) (Krings et al., 2006; You et al., 2020). Furthermore, this induction works regardless of whether the glucose is present or not (Krings et al., 2006; Klaffl et al., 2013). Besides, compared with IPTG, myo-inositol is much cheaper and non-toxic to cells (Krings et al., 2006; Takeno et al., 2016). Above all, myo-inositol seems an alternative inducer for gene expression in *C. glutamicum*.

In this study, a myo-inositol-inducible expression vector pMI-4 for *C. glutamicum* was constructed. Meanwhile, the chassis strain that is suitable for maintaining pMI-4 and unable to consume myo-inositol, *C. glutamicum*Δ*iolG*Δ*oxiC*Δ*oxiD*Δ*oxiE*, was developed. This constructed expression system was successfully applied to express *hemA* ^*M*^ (encoding glutamyl-tRNA reductase) and *hemL* (encoding glutamate-1-semialdehyde aminotransferase) for production of 5-aminolevulinic acid (5-ALA).

## Materials and Methods

### Bacterial Strains, Media, and Cultivation Conditions

The strains and plasmids used are listed in Table 1. *Escherichia coli*, used for recombinant DNA experiments, was cultured in lysogeny broth medium at 37°C. *C. glutamicum* ATCC 13032 and its recombinants were cultured at 30°C in Brain Heart Infusion (BHI) medium or CGXII medium. Modified CGXII medium [50 g/l glucose, 15 g/L (NH_4_)_2_SO_4_, 2 g/l yeast extract, 4 g/l tryptone, 1 g/l KH_2_PO_4_, 0.25 g/l MgSO_4_·7H_2_O, 42 g/l 3-morpholinopropanesulfonic acid, 10 mg/l CaCl_2_, 0.1 mg/l FeSO_4_·7H_2_O, 0.1 mg/l MnSO_4_·7H_2_O, 1 mg/l ZnSO_4_·7H_2_O, 0.2 mg/l CuSO_4_, 0.02 mg/l NiCl_2_·6H_2_O, and 0.2 mg/l biotin] was used for production of 5-ALA. When necessary, media were supplemented with 20 μg/ml chloramphenicol or 40 μg/ml kanamycin.

**TABLE 1:** Strains and plasmids used in this study.

Strains/plasmids	Characteristics	Source
Strains		
*Escherichia coli* DH5α	F-, Δ(*lacZYA-argF*)U169 *recA1endA1 hsdR17*	Purchased from Invitrogen
*Corynebacterium glutamicum* ATCC 13032	Wild type	Laboratory stock
*C. glutamicum*/pMI-1-*gfp*	*C. glutamicum* ATCC 13032 harboring pMI-1-*gfp*	This work
*C. glutamicum*/pMI-3-*gfp*	*C. glutamicum* ATCC 13032 harboring pMI-3-*gfp*	This work
*C. glutamicum*/pMI-4-*gfp*	*C. glutamicum* ATCC 13032 harboring pMI-4-*gfp*	This work
*C. glutamicum*Δ*iolG* Δ*oxiC*Δ*oxiD*Δ*oxiE*	*C. glutamicum* ATCC 13032 in which Δ*iolG* and Δ*oxiC*-Δ*oxiD*-Δ*oxiE* cluster were deleted	This work
*C. glutamicum*Δ*iolG*Δ*oxiC*Δ*oxiD*Δ*oxiE/*pMI-4-*gfp*	*C. glutamicum*Δ*iolG*Δ*oxiC*Δ*oxiD*Δ*oxiE* harboring pMI-4-*gfp*	This work
AL-1	Native promoters of *ppc* and *gltA* were replaced by P_*tuf*_ in *C. glutamicum*Δ*iolG*Δ*oxiC*Δ*oxiD*Δ*oxiE*	This work
AL-CK1	AL-1 harboring pMI-4	This work
AL-2	AL-1 harboring pMI-4-*hemA* ^*M*^-*hemL*	This work
AL-CK2	AL-1 harboring pAL1	This work
AL-3	AL-2 with a copy of *pntAB* driven by P_*iolT1*_ in genome (*cgl 2000*:P_*iolT1*_-*pntAB*)	This work
Plasmids		
pXMJ19	Cm^r^, P_*tac*_-MCS	Jakoby et al. (1999)
pEGFP-N1	Kan^r^, *gfp*	Laboratory stock
pK18*mobsacB*	Integration vector, Kan^r^, *sacB*	Schäfer, et al. (1994)
pMI-1	pXMJ19Δ*lacI*ΔP_*tac*_	This work
pMI-2	pMI-1 with P_*iolT1*_	This work
pMI-3	pMI-2 with *iolR* cassette	This work
pMI-4	pMI-2 with *iolR* cassette driven by P_*tuf*_	This work
pMI-3-*gfp*	pMI-3 harboring *gfp*	This work
pMI-4-*gfp*	pMI-4 harboring *gfp*	This work
pAL1	pXMJ19 harboring *hemA* ^*M*^ and *hemL* located tandemly in one operon	Zhang et al. (2020)
pMI-4-*hemA* ^*M*^-*hemL*	pMI-4 harboring *hemA* ^*M*^ and *hemL* located tandemly in one operon	This work
pK18*mobsacB*Δ*iolG*	pK18*mobsacB* harboring upstream homologous arm (UHA) and downstream homologous arm (DHA) of *iolG*	This work
pK18*mobsacB*Δ*oxiII*	pK18*mobsacB* harboring UHA and DHA of Δ*oxiC*-Δ*oxiD*-Δ*oxiE* cluster	This work
pK18*mobsacB*P_*tuf*_:P_*ppc*_	pK18*mobsacB* harboring P_*ppc*_ UHA, P_*tuf*_, and P_*ppc*_ DHA	Zhang et al. (2018)
pK18*mobsacB*P_*tuf*_:P_*gltA*_	pK18*mobsacB* harboring P_*gltA*_ UHA, P_*tuf*_, and P_*gltA*_ DHA	Zhang et al. (2018)
pK18*mobsacBcgl 2000*:P_*iolT1*_-*pntAB*	pK18*mobsacB* harboring UHA and DHA of *cgl 2000* and P_*iolT1*_-*pntAB*	This work

To investigate the effect of myo-inositol on cell growth, *C. glutamicum* ATCC 13032 cells were cultivated in CGXII containing 0.1–100 mM of myo-inositol, and the optical density at 600 nm (OD_600_) was determined after cultivation of 48 h at 30°C with shaking at 200 rpm. To investigate the effect of myo-inositol on glucose utilization, *C. glutamicum*Δ*iolG*Δ*oxiC*Δ*oxiD*Δ*oxiE* cells were cultivated in CGXII medium containing 5 mM of myo-inositol or not at 30°C with shaking at 200 rpm. The final concentration of glucose was determined after cultivation of 48 h.

### Construction of Strains and Plasmids

All the primers used in this study are listed in Supplementary Table S1. *E. coli* DH5α was used for vector construction experiments. *C. glutamicum* ATCC 13032 was used as a base strain for investigating the characteristics of plasmids and for construction of the non-myo-inositol-degrading host. Preparation of competent cells and transformation were performed according to the standard protocols (Sambrook and Russell, 2001; Eggeling and Bott, 2005). Strains and plasmids were constructed as follows.

The plasmid pXMJ19 was used as the backbone. The fragments amplified from pXMJ19 with primers PX-1 and PX-2 were digested by *Hpa* I and were subsequently self-ligated to obtain pMI-1. P_*iolT1*_ was amplified with primers PX-3 and PX-4 from genomic DNA of *C. glutamicum* ATCC 13032, and the resulting fragments were ligated to *Hpa* I-digested pMI-1 using a ClonExpress II One Step Cloning Kit, generating pMI-2. Similarly, fragments of *iolR* cassette amplified with primers PX-5 and PX-6 were ligated to pMI-2, resulting in pMI-3. Primers PX-7/tuf-iolR-1 and tuf-iolR-2/PX-6 were used to amplify *iolR* cassette (without the native promoter) and the constitutive promoter P_*tuf*_. The fragments were fused using overlap PCR and then were ligated to pMI-2 to obtain pMI-4. Primers gfp-1 and gfp-2 were used to amplify *gfp* from vector pEGFP-N1, which was ligated into *Hind* III-digested pMI-3 and pMI-4, resulting in pMI-3-*gfp* and pMI-4-*gfp*, respectively. The fused *hemA* ^*M*^-*hemL* was amplified with primers PAL-1 and PAL-2 from pAL1 (Zhang et al., 2020) and was ligated into *Hind* III-digested pMI-4, resulting in pMI-4-*hemA* ^*M*^-*hemL*.

Primers iolG-1/iolG-2 and iolG-3/iolG-4 were used to amplify the upstream homologous arm and downstream homologous arm of *iolG* from *C. glutamicum* ATCC 13032. The fragments were fused using overlap PCR, and the resulting fragments were ligated to *Xba* I/*Eco*R I-digested pK18mob*sacB*, generating pK18*mobsacB*Δ*iolG*; similarly, pK18*mobsacB*Δ*oxiII* for deleting *oxiC*-*oxiD*-*oxiE* cluster as well as pK18*mobsacBcgl 2000*: P_*iolT1*_-*pntAB* for *pntAB* integration were constructed. Knockout of *iolG* and *oxiC*-*oxiD*-*oxiE* cluster occurred *via* two successive recombination events using pK18*mobsacB*Δ*iolG* and pK18*mobsacB*Δ*oxiII* to generate *C. glutamicum*Δ*iolG*Δ*oxiC*Δ*oxiD*Δ*oxiE*. AL-1 and AL-3 were constructed using the same approach.

The plasmids pMI-3-*gfp* and pMI-4-*gfp* were transformed into *C. glutamicum* ATCC 13032, and pMI-4-*hemA* ^*M*^-*hemL* was transformed into AL-1, obtaining *C. glutamicum*/pMI-3-*gfp*, *C. glutamicum*/pMI-4-*gfp*, and AL-2, respectively.

### Stability Analysis of pMI-4

*C. glutamicum*Δ*iolG*Δ*oxiC*Δ*oxiD*Δ*oxiE*/pMI-4 and *C. glutamicum*Δ*iolG*Δ*oxiC*Δ*oxiD*Δ*oxiE*/pXMJ19 were respectively inoculated in BHI medium containing chloramphenicol and were cultivated overnight at 30°C with shaking at 200 rpm as the seed. The seed was then inoculated into BHI medium without chloramphenicol and was cultivated at 30°C for 30 generations. Samples were taken every five generations. After appropriate dilution, they were spread on BHI plates with or without chloramphenicol, followed by incubation for 24 h. The ratio of colonies on BHI with chloramphenicol/colonies on BHI without chloramphenicol was used to determine the stability.

### Conditions for 5-ALA Production

A single colony was transferred to 5 ml BHI medium and was cultivated overnight at 30°C with shaking at 200 rpm. Next, the culture was inoculated into a 500-ml shake flask containing 45 ml of modified CGXII medium for a 64-h incubation at 30°C, 200 rpm. To induce gene expression, myo-inositol was added at a final concentration of 0.1–5 mM when the OD_600_ of cell reached approximately 0.8.

### Detection of GFP Activity

*C. glutamicum*/pMI-1-*gfp*, *C. glutamicum*/pMI-3-*gfp*, and *C. glutamicum*/pMI-4-*gfp* were respectively grown overnight in 24-deep-well plates containing 2 ml/well CGXII medium. Then, 20 μl of each culture were inoculated into 2 ml fresh CGXII medium and grown for 24 h. Fluorescence intensities normalized against culture OD_600_ (determined using a Synergy H4 Microplate Reader, BioTek, United States) were used to indicate the expression level of GFP (Peng et al., 2017).

### Real-Time Quantitative PCR

The transcriptional level of *gfp*, *hemA* ^*M*^, and *pntAB* was quantified using real-time quantitative PCR. Total RNA was extracted after 4 h of induction by myo-inositol using RNAiso Plus (Takara Bio Inc., Dalian, China), and cDNA was obtained by reverse transcription with a PrimeScript RT Reagent Kit (Takara Bio Inc., Dalian, China). Gene transcription levels were measured using SYBR Premix Ex TaqTM II (Takara Bio Inc., Dalian, China), and 16S rDNA was used as an internal control. Data were analyzed using the 2^−ΔΔCT^ method (Livak and Schmittgen, 2001).

### Analytical Procedure

Cell growth was monitored by measuring the OD_600_. Concentrations of 5-ALA were measured with modified Ehrlich’s reagent (Mauzerall and Granick, 1956). Briefly, the supernatants of cultures were reacted with acetylacetone (in sodium acetate buffer, pH 4.8, 1.0 M) at 80°C for 20 min. The absorbance at 553 nm was measured 30 min after adding of the modified Ehrlich’s reagent. *C. glutamicum* cells grown in CGXII medium for 24 h were harvested by centrifugation at 4°C and 13,000×*g* for 10 min. The intracellular NADPH was quantified using an Enzychrom NADP^+^/NADPH assay kit, following the manufacturer’s instruction. The myo-inositol concentrations were determined by HPLC (Bio-Rad Aminex HPX-87H column) with 5 mM H_2_SO_4_ as the mobile phase and a refractive index detector (Lu et al., 2018).

### Statistical Analysis

Statistical significance was determined by one-way analysis of variance, followed by Dunnett’s multiple comparison test. The data are average of three biological replicates with error bars representing standard deviation. Results with *p* values of less than or equal to 0.05 were considered significant.

## Results and Discussion

### Construction of Myo-Inositol-Inducing Expression Vector pMI-4

**FIGURE 2:**
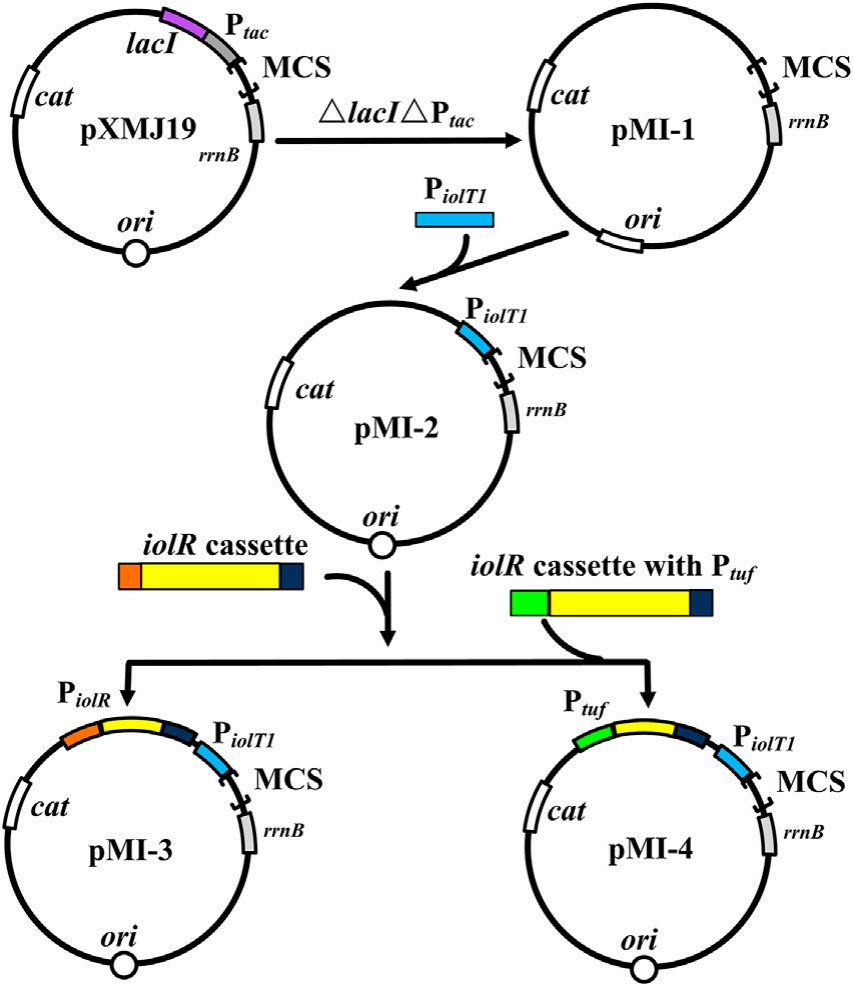
Construction of pMI-4. MCS, multiple cloning sites; *cat*, chloramphenicol resistance gene; *rrnB*, the transcriptional terminator; P_*tac*_, *tac* promoter; *lacI*, repressor LacI of P_*tac*_-encoding gene; *ori*, the origin of replication for *C. glutamicum*; P_*iolT1*_, *iolT1* promoter; *iolR* cassette, fragment comprises promoter, encoding sequence, and terminator of *iolR*; P_*tuf*_, promoter of the gene encoding elongation factor TU.

To verify the non-toxicity of myo-inositol to cell growth, *C. glutamicum* ATCC 13032 cells were cultivated in a medium containing different concentrations of myo-inositol (0.1–100 mM). The biomass of the cells under each concentration of myo-inositol exhibited no difference and was similar with those in the medium without myo-inositol (Supplementary Figure S1), indicating the non-toxicity of myo-inositol to cell growth. The myo-inositol-inducing vector pMI-4 was constructed for use in *C. glutamicum* as shown in Figure 2. The plasmid pXMJ19, carrying the chloramphenicol resistance gene *cat* and the inducible promoter P_*tac*_, was used as the backbone (Jakoby et al., 1999). Herein, P_*iolT1*_ of *C. glutamicum*, the promoter of myo-inositol transport protein encoding gene *iolT1*, was used. P_*iolT1*_ is a myo-inositol-inducing promoter, which is repressed by the repressor IolR (encoded by *iolR*) in the absence of myo-inositol, but was active in the presence of myo-inositol (Figure 1). Initially, the P_*tac*_ and *lacI* ^q^ cassette were removed from pXMJ19, generating pMI-1 (5,281 bp). Subsequently, the P_*iolT1*_ was introduced to the upstream of multi-cloning sites in pMI-1, resulting in pMI-2 (5,488 bp). Finally, the *iolR* cassette from *C. glutamicum* ATCC 13032 was introduced to the upstream of P_*iolT1*_ in pMI-2, resulting in pMI-3 (6,750 bp).

**FIGURE 3:**
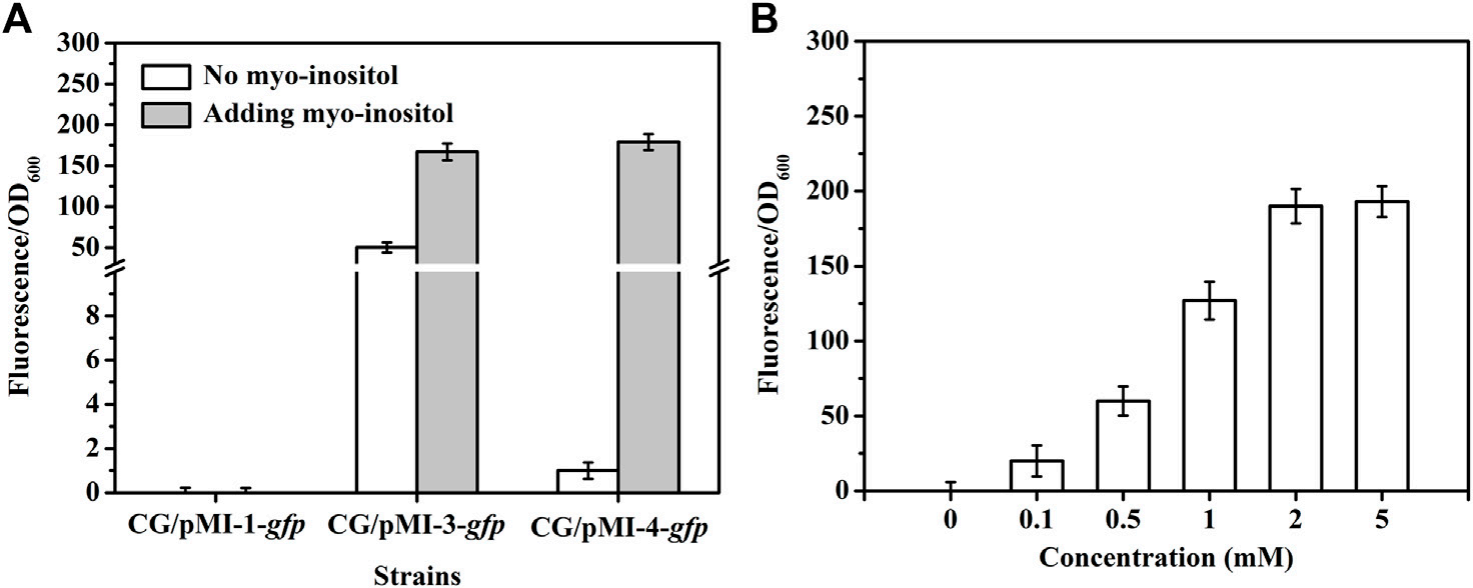
GFP fluorescence intensities in *C. glutamicum* strains. **(A)** GFP fluorescence intensities in *C. glutamicum* strains harboring different vectors in the absence (white) or presence of myo-inositol (gray). CG/pMI-1-*gfp*, CG/pMI-3-*gfp*, and CG/pMI-4-*gfp* indicate *C. glutamicum*/pMI-1-*gfp*, *C. glutamicum*/pMI-3-*gfp*, and *C. glutamicum*/pMI-4-*gfp*, respectively. *C. glutamicum*/pMI-1-*gfp* was used as a control. In this strain, GFP was not expressed because pMI-1-*gfp* lacks a promoter. The vector pMI-3-*gfp* contains P_*iolT1*_ and *iolR* cassette with its native promoter P_*iolR*_, and the vector pMI-4-*gfp* contains P_*iolT1*_ and *iolR* ^*q*^ cassette with the constitutive P_*tuf*_. **(B)** The effect of myo-inositol concentrations on GFP expression in *C. glutamicum*/pMI-4-*gfp*. *C. glutamicum*/pMI-4-*gfp* was induced by myo-inositol at different concentrations.

In order to investigate whether pMI-3 was induced by myo-inositol, the *gfp* gene encoding green fluorescent protein was introduced to pMI-1 and pMI-3, resulting in pMI-1-*gfp* (as a control) and pMI-3-*gfp*, respectively. These two plasmids were respectively transformed into *C. glutamicum* ATCC 13032 to generate *C. glutamicum*/pMI-1-*gfp* and *C. glutamicum*/pMI-3-*gfp*. The GFP activities in *C. glutamicum*/pMI-1-*gfp* and *C. glutamicum*/pMI-3-*gfp* were determined. As shown in Figure 3A, GFP activity was hardly detected in the control strain *C. glutamicum*/pMI-1-*gfp* neither in the presence nor the absence of myo-inositol. In contrast, *C. glutamicum*/pMI-3-*gfp* exhibited significant GFP fluorescence (fluorescence/OD_600_, 167.1) in the presence of myo-inositol, indicating that pMI-3 was myo-inositol inducible.

Notably, considerable GFP fluorescence of *C. glutamicum*/pMI-3-*gfp* was also observed in the absence of myo-inositol (fluorescence/OD_600_, 50.3), indicating the leaky expression of GFP. It was probably due to the negative auto-regulation of *iolR* gene (Krings et al., 2006; Klaffl et al., 2013). To verify our hypothesis, the native promoter in *iolR* cassette was replaced with the constitutive promoter *tuf* to generate *iolR* ^*q*^ cassette, which was then inserted into pMI-2 (resulting in pMI-4, 6,643 bp). The *gfp* gene was introduced to pMI-4, generating pMI-4-*gfp*, and was subsequently transformed into *C. glutamicum* ATCC 13032 to generate *C. glutamicum*/pMI-4-*gfp*. As shown in Figure 3A, GFP of *C. glutamicum*/pMI-4-*gfp* was only active in the presence of myo-inositol (fluorescence/OD_600_, 179.3). This result indicated the non-leaky expression of GFP in the pMI-4-*gfp* with *iolR* ^*q*^.

To further investigate the characteristics of pMI-4, the effect of myo-inositol concentration on GFP expression in *C. glutamicum*/pMI-4-*gfp* was determined. As shown inFigure 3B, the GFP fluorescence intensities were reinforced with the increase of myo-inositol concentration and then reached a constant level (approximately 190), indicating the dose-dependent effects of myo-inositol on GFP expression when the myo-inositol concentration was below 2 mM.

**FIGURE 4:**
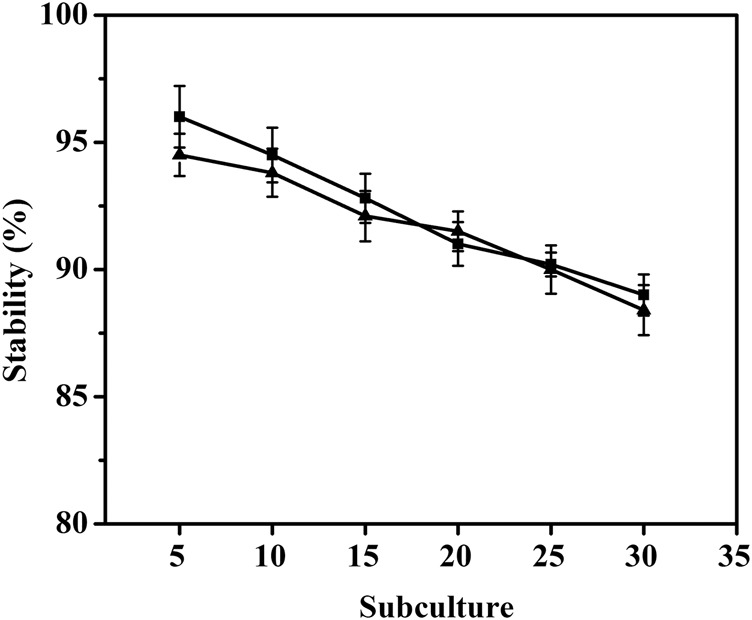
Stability of the vector pMI-4. Filled square (■) represents *C. glutamicum*/pMI-4, and filled triangle (▲) represents *C. glutamicum*/pXMJ19.

To investigate the stability of pMI-4 in *C. glutamicum*, it was transformed into *C. glutamicum* ATCC 13032 to generate *C. glutamicum*/pMI-4, and *C. glutamicum*/pXMJ19 was used a control. As shown in Figure 4, no significant difference in the stability of the vectors pMI-4 (approximately 96%–88%) and pXMJ19 was observed after 30 generations without chloramphenicol.

### Blocking Myo-Inositol Degradation Pathway in *C. glutamicum*

**FIGURE 5:**
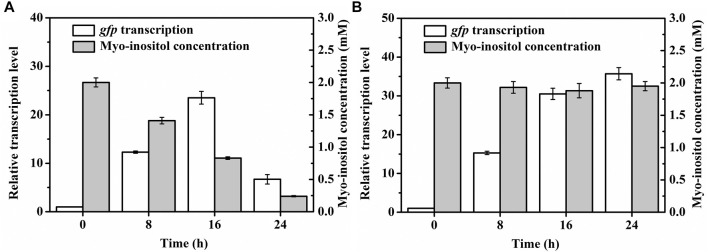
Transcriptional levels of *gfp* in *C. glutamicum* strains during the cultivation process. **(A)** Transcriptional levels of *gfp* in *C. glutamicum*/pMI-4-*gfp* and the myo-inositol concentrations during the cultivation process. **(B)** Transcriptional levels of *gfp* in *C. glutamicum*Δ*iolG*Δ*oxiC*Δ*oxiD*Δ*oxiE*/pMI-4-*gfp* and the myo-inositol concentrations during the cultivation process.

To further test the applicability of pMI-4, the transcriptional level of *gfp* in *C. glutamicum*/pMI-4-*gfp* was detected during the fermentation. As shown in Figure 5A, the transcriptional level of *gfp* was gradually increased during the first 16 h of fermentation, but sharply declined after then. It was supposed that myo-inositol was utilized by cells, and the lowered concentration resulted in the recovery of IolR depression (Krings et al., 2006). Then, the concentration of myo-inositol was detected, and its consumption was found to start at 8 h (Figure 5A). Therefore, it was essential to block the degradation of myo-inositol to maintain its concentration at a constant level.

**FIGURE 6:**
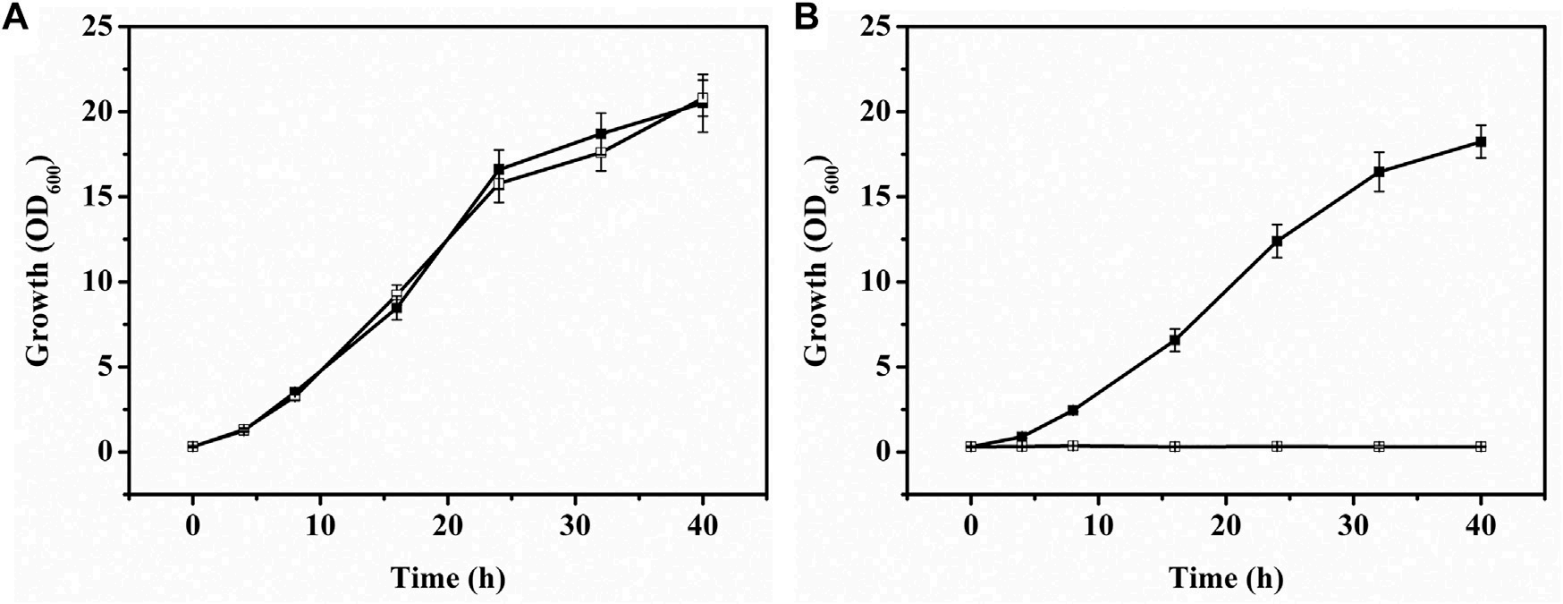
Growth of *C. glutamicum* strains on glucose or myo-inositol. **(A)** Growth of *C. glutamicum* ATCC 13032 [filled square (■)] and *C. glutamicum*Δ*iolG*Δ*oxiC*Δ*oxiD*Δ*oxiE* [white square (□)] on glucose. **(B)** Growth of *C. glutamicum* ATCC 13032 [filled square (■)] and *C. glutamicum*Δ*iolG*Δ*oxiC*Δ*oxiD*Δ*oxiE* [white square (□)] on myo-inositol.

Myo-inositol dehydrogenase is the first enzyme responsible for degradation of myo-inositol (Krings et al., 2006). There are four myo-inositol dehydrogenase isozymes (encoded by *iolG*, *oxiC*, *oxiD*, and *oxiE*, respectively) in *C. glutamicum*. These four genes were deleted in *C. glutamicum* ATCC 13032. The generated *C. glutamicum*Δ*iolG*Δ*oxiC*Δ*oxiD*Δ*oxiE* was cultivated on CGXII containing myo-inositol (20 g/l) as carbon source to investigate its ability of myo-inositol utilization. As shown in Figure 6, unlike the wild-type *C. glutamicum* ATCC13032, *C. glutamicum*Δ*iolG*Δ*oxiC*Δ*oxiD*Δ*oxiE* was not able to grow on myo-inositol. Whereas, its growth on glucose was hardly affected compared with *C. glutamicum* ATCC13032, indicating that deleting the myo-inositol dehydrogenase isozymes resulted in the disability of myo-inositol degradation but did not affect the growth on glucose. To investigate the effect of myo-inositol on glucose utilization by *C. glutamicum*Δ*iolG*Δ*oxiC*Δ*oxiD*Δ*oxiE*, the cells were cultivated in CGXII medium containing 5 mM of myo-inositol (the maximum dose for induction). No significant differences in cell growth and glucose consumption were observed between the cells cultivated with and without myo-inositol (Supplementary Figure S2). So, it was inferred that glucose metabolism may not be negatively affected under the myo-inositol concentration for induction.

The plasmid pMI-4-*gfp* was transformed into the *C. glutamicum*Δ*iolG*Δ*oxiC*Δ*oxiD*Δ*oxiE*, and the consumption of myo-inositol as well as the transcription level of *gfp* was determined. As shown in Figure 5B, no consumption of myo-inositol by *C. glutamicum*Δ*iolG*Δ*oxiC*Δ*oxiD*Δ*oxiE/*pMI-4-*gfp* was detected during the cultivation process. Meanwhile, a continuous increase in transcriptional levels of *gfp* was observed in this strain. These results further indicated that the degradation of myo-inositol was successfully blocked by deleting the myo-inositol dehydrogenase-encoding genes, and this resulted in constantly reinforced transcriptional level of *gfp*.

### Application of the Myo-Inositol-Inducing Expression System for Producing 5-ALA

To investigate the application of the myo-inositol-inducing expression system, it was used to overexpress the key genes for production of 5-ALA. Initially, to enhance the α-ketoglutarate supply for the synthesis of 5-ALA, the *ppc* (encoding phosphoenolpyruvate carboxylase) and *gltA* (encoding citrate synthase) in *C. glutamicum*Δ*iolG*Δ*oxiC*Δ*oxiD*Δ*oxiE* was overexpressed by replacing the native promoters with the constitutive promoter *tuf*, resulting in AL-1. The glutamyl-tRNA reductase (encoded by *hemA*) and glutamate-1-semialdehyde aminotransferase (encoded by *hemL*) are rate-limiting enzymes for 5-ALA synthesis (Yu et al., 2015). The mutant *hemA* ^*M*^ (two Lys residues inserted between Thr-2 and Leu-3) from *Salmonella arizona* (Yu et al., 2015) and *hemL* from *E. coli* were proven conducive for efficient production of 5-ALA. Therefore, these two genes were introduced into pMI-4. The generated pMI-4-*hemA* ^*M*^-*hemL* was subsequently transformed into AL-1, resulting in AL-2. At the same time, pMI-4 and pAL1 (pXMJ19 harboring *hemA* ^*M*^ and *hemL*) was transformed into AL-1 to obtain AL-CK1 and AL-CK2, which were used as controls.

**FIGURE 7:**
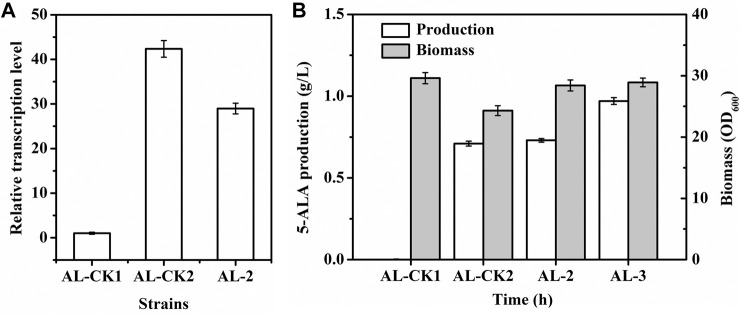
5-ALA production, biomass, and *hemA* ^*M*^ transcription of strains. **(A)** *hemA* ^*M*^ transcription; **(B)** 5-ALA production (white) and biomass (gray). AL-CK1 was used as a control. AL-CK1, AL-1 (*C. glutamicum*Δ*iolG*Δ*oxiC*Δ*oxiD*Δ*oxiE*P_*ppc*_:P_*tuf*_P_*gltA*_:P_*tuf*_) harboring pMI-4; AL-CK2, AL-1 harboring pAL1; AL-2, AL-1 harboring pMI-4-*hemA* ^*M*^-*hemL*; AL-3, *C. glutamicum*Δ*iolG*Δ*oxiC*Δ*oxiD*Δ*oxiE*P_*ppc*_:P_*tuf*_P_*gltA*_:P_*tuf*_ *cgl 2000*:P_*iolT1*_-*pntAB* harboring pMI-4-*hemA* ^*M*^-*hemL*.

Fermentation was performed in flasks to evaluate the characteristics of AL-2 and then the performance of this myo-inositol-inducing expression system. Compared with the transcriptional level of *hemA* ^*M*^ (approximately 42-fold) in AL-CK2 induced by IPTG (1 mM), the transcriptional level of *hemA* ^*M*^ in AL-2 induced by the same dose of myo-inositol was lower (approximately 29-fold, Figure 7A). Nevertheless, the biomass of AL-2 (OD_600_ = 28.4) was significantly higher than that of AL-CK2 (OD_600_ = 24.3, Figure 7B). Furthermore, the final production of 5-ALA by AL-2 reached 0.73 g/l, slightly higher (4.2%) than that by AL-CK2 (0.71 g/l, Figure 7B). These results indicated that a stronger expression of genes sometimes was not essential for efficient production. Notably, the glucose consumption by AL-2 (30.1 g/l) was 5.6% lower than that by AL-CK2 (31.9 g/l), and this generated an increase of 9.0% in the yield of 5-ALA (24.3 VS 22.3 mg/g glucose). It was supposed that the increased cell growth and decreased glucose consumption were due to the following reasons: unlike IPTG, myo-inositol was not toxic to cell, and thus, cell growth and metabolism were not inhibited; furthermore, the constructed myo-inositol-inducible vector relieved the metabolic burden in some degree since the transcription intensity of P_*iolT1*_ was lower than that of P_*tac*_ in pXMJ19 under the same dose of inducer, which resulted in relatively lower glucose requirement and improved cell growth.

The glutamyl-tRNA reductase is NADPH dependent, and enhancing NADPH regeneration has been proven to facilitate increased 5-ALA production (Zhang et al., 2020). Previous reports showed that overexpressing the *pntAB* gene (encoding pyridine nucleotide transhydrogenase) could improve NADPH regeneration (Kabus et al., 2007; Hao et al., 2020). To test the applicability of P_*iolT1*_, a copy of *pntAB* driven by P_*iolT1*_ was integrated into the genome of AL-2 (generating AL-3) so that transcription of *pntAB* was induced by myo-inositol. As a result, the transcription level of *pntAB* in AL-3 was detected to be gradually enhanced after the addition of myo-inositol and maintained constant after 16 h (Supplementary Figure S3). Moreover, the intracellular NADPH in AL-3 was significantly improved by 27.7% (from 0.65 to 0.83 μmol/g DCW). As shown in Figure 7A, the production of 5-ALA by AL-3 was increased by 32.9% (0.97 g/l) compared with AL-2. This result indicated the potential application of P_*iolT1*_ in metabolic engineering.

Besides *iolT1*, transcription of several genes, such as *iolA* (encoding aldehyde dehydrogenase) and *iolC* (encoding carbohydrate kinase), was detected up-regulated to different levels when *C. glutamicum* cells were grown on myo-inositol. These results indicated that the promoters of these genes are probably inducible by myo-inositol, and strengths of these promoters are diverse. Therefore, these promoters will be exploited for gene overexpression in the future work.

## Conclusion

A myo-inositol-inducible expression vector pMI-4, containing the *iolR* ^*q*^ cassette and P_*iolT1*_, was constructed in this study. The pMI-4 was proven to stably replicate in *C. glutamicum* cells without antibiotic selection pressure. Furthermore, the chassis *C. glutamicumΔiolGΔoxiCΔoxiDΔoxiE*, which was not able to degrade myo-inositol and suitable for maintaining pMI-4, was developed as the host strain. Through overexpression of *hemA* ^*M*^ and *hemL* using the vector pMI-4 in the engineered chassis *C. glutamicumΔiolGΔoxiCΔoxiDΔoxiE*, the production of 5-ALA achieved 0.73 g/l, which indicated the potential application of this expression system in metabolic engineering and industrial production. Furthermore, the P_*iolT1*_ was proved to be applicable for overexpressing genes by integrating to genome.

## Data Availability

The original contributions presented in the study are included in the article/Supplementary Material, and further inquiries can be directed to the corresponding authors.
