# Supplementary material for: A Myo-Inositol-Inducible Expression System for Corynebacterium glutamicum and Its Application
Source: Front Bioeng Biotechnol. 2021 Nov 15;9:746322. doi: 10.3389/fbioe.2021.746322 (PMC8634428; doi:10.3389/fbioe.2021.746322)
Supplement: Supplementary file 1 [file DataSheet1.docx]

**Supplementary Materials**

**Figure Captions**

**Figure S1 Effect of myo-inositol on growth of *C. glutamicum* ATCC 13032.**

**Figure S2 Influence of myo-inositol on growth and glucose consumption of *C. glutamicum*Δ*iolG*Δ*oxiC*Δ*oxiD*Δ*oxiE*.**

(-) represents *C. glutamicum*Δ*iolG*Δ*oxiC*Δ*oxiD*Δ*oxiE* grown in CGXII without myo-inositol; (+) represents *C. glutamicum*Δ*iolG*Δ*oxiC*Δ*oxiD*Δ*oxiE* grown in CGXII with 5 mM myo-inositol.

**Figure S3 Transcriptional levels of *pntAB* in AL-3.**

**

**

**Figure S1 Effect of myo-inositol on growth of *C. glutamicum* ATCC 13032**





**Figure S2 Influence of myo-inositol on growth and glucose consumption of *C. glutamicum*Δ*iolG*Δ*oxiC*Δ*oxiD*Δ*oxiE***





**Figure S3 Transcriptional levels of *pntAB* in AL-3**

**Supplementary Tables**

**Table S1 Primers used in this study**

| Primers | Sequences (5’-3’) |
| --- | --- |
| PX-1 | CCG*GTTAAC*AATTAAGCTTGCATGCCTGCA* |
| PX-1 | CCG*GTTAAC*agggcaatcagctgttgcccg |
| PX-3 | ACAGCTGATTGCCCT*GTTAAC*ATGGAAAACCATACCCAAGCAG |
| PX-4 | TGCAGGCATGCAAGCTTAATTCTTGTCTCCTAAGTTTGTCGTGCC |
| PX-5 | ACAGCTGATTGCCCTACCCTCACGATCGCATGTCA |
| PX-6 | CGGGTTCAGGGGGTGCTTTCACTCGCTCTCCATCCG |
| gfp-1 | CACAGGAAACAGAATTATGGTGAGCAAGGGCGAGG |
| gfp-2 | gcatgcctgcaggtcgactcTTACTTGTACAGCTCGTCCATGC |
| PX-7 | ACAGCTGATTGCCCTAGCGTGTCAGTAGGCGCGT |
| tuf-iolR-1 | TGGGAGCTTCGGTGGTCATTGTATGTCCTCCTGGACTTCGTG |
| tuf-iolR-2 | CACGAAGTCCAGGAGGACATACAATGACCACCGAAGCTCCCA |
| PAL-1 | cacaggaaacagaattaattAAGCTTAGAAAGGTGTGTTTCACCCATGA |
| PAL-2 | GTACCCGGGGATCCTCTAGATCACAACTTCGCAAACACCC |
| iolG-1 | ACGACGGCCAGTGCCAAGCTTCCGGTCACGACCGCCT |
| iolG-2 | ATACGAACCACGTTCAAAACTCCTTAGTTGACGCGTGCGAAGTG |
| iolG-3 | CACTTCGCACGCGTCAACTAAGGAGTTTTGAACGTGGTTCGTAT |
| iolG-4 | GAGCTCGGTACCCGGGGATCCTTATGACTCGCCATGCTTCAATAC |
| ioxII-1 | ACGACGGCCAGTGCCAAGCTTGGATCGCCGCTGTAGGAGCAC |
| ioxII-2 | CAGCGCACGCACCTCAACTGGCATTTTTTGATCACTCATGGGAATTCT |
| ioxII-3 | AGAATTCCCATGAGTGATCAAAAAATGCCAGTTGAGGTGCGTGCGCTG |
| ioxII-4 | GAGCTCGGTACCCGGGGATCCTTGGTTAGGCAGGATGAGGTTGAGA |
| pntAB-1 | ACGACGGCCAGTGCCAAGCTTGTGTTCTATCCTGGCGGCAT |
| pntAB-2 | ACGCGCCTACTGACACGCTACCAAATAAGAGCGTTGGGATTA |
| pntAB-3 | TAATCCCAACGCTCTTATTTGGTAGCGTGTCAGTAGGCGCGT |
| pntAB-4 | CTCTTGGTATGCCAATTCGCATTGTATGTCCTCCTGGACTTCGT |
| pntAB-5 | CACGAAGTCCAGGAGGACATACAATGCGAATTGGCATACCAAGAG |
| pntAB-6 | TGCGTGGAGCGCTTAGAACTGATGCAATCCTGAAAGCTCTGTAA |
| pntAB-7 | TTACAGAGCTTTCAGGATTGCATCAGTTCTAAGCGCTCCACGCA |
| pntAB-8 | GAGCTCGGTACCCGGGGATCCTTTTGAAAACCAACCTATGATCCTCG |
| 16S-F | AGAACCACCGCCTGC TCACC |
| 16S-R | CCGTCGTCGTAGTTGTACTCCTTG |
| gfp-F | CAGTGCTTCAGCCGCTACCC |
| gfp -R | AGCTCGATGCGGTTCACCAG |
| hemA-F | ATCGCCAACCGTACCCG |
| hemA-R | GGCACGCTCCACCATACCT |
| pntAB-F | TCTGCCGACGCAATCCTCACA |
| pntAB-R | CCGCGAATCACCACATCATCAAA |

* Italics indicate the restriction enzyme sites; Underlined letters indicate the sequence homogenous to the plasmids
